# Medical provision and urban-rural differences in maternal mortality in late nineteenth century Scotland

**DOI:** 10.1016/j.socscimed.2018.01.028

**Published:** 2018-03

**Authors:** Alice Reid, Eilidh Garrett

**Affiliations:** a Department of Geography, University of Cambridge, UK; b Department of History, University of Essex, UK

**Keywords:** Scotland, Maternal mortality, Doctors, 19th century, Certification of death, Under-recording, Obstetric transition

## Abstract

This paper examines the effect of variable reporting and coding practices on the measurement of maternal mortality in urban and rural Scotland, 1861–1901, using recorded causes of death and women who died within six weeks of childbirth. This setting provides data (n = 604 maternal deaths) to compare maternal mortality identified by cause of death with maternal mortality identified by record linkage and to contrast urban and rural settings with different certification practices. We find that underreporting was most significant for indirect causes, and that indirect causes accounted for a high proportion of maternal mortality where the infectious disease load was high. However, distinguishing between indirect and direct maternal mortality can be problematic even where cause of death reporting appears accurate. Paradoxically, underreporting of maternal deaths was higher in urban areas where deaths were routinely certified by doctors, and we argue that where there are significant differences in medical provision and reported deaths, differences in maternal mortality may reflect certification practices as much as true differences. Better health services might therefore give the impression that maternal mortality was lower than it actually was. We end with reflections on the interpretation of maternal mortality statistics and implications for the concept of the obstetric transition.

## Highlights

• There was marked under-reporting of maternal mortality in 19th century Scotland.
• Differences in maternal mortality may reflect different certification practices.
• Doctors were more likely to mis-report maternal deaths than lay informants.
• Maternal mortality may not be a good indicator of the coverage of health services.
• Conceptual models relying on cause of death should be treated with caution.

## 1 Introduction

After centuries of relative stability, almost all demographic regimes have undergone major changes in the last 150 years. The nearly universal fall from high and fluctuating birth and death rates to low rates of both has been designated as the demographic transition (Notestein, 1945). Relatedly, the epidemiologic transition describes a change in the balance of causes of death as mortality falls, from a dominance of infectious disease when mortality is high to a dominance of chronic and non-communicable diseases when mortality is low (Omran, 1971). Other transitions have also been proposed, including the health transition (Frenk et al., 1991), the nutritional transition (Popkin, 1993) and the obstetric transition (Souza et al., 2014). The obstetric transition describes the changes that take place as maternal mortality declines, encapsulating a shift in the balance of maternal mortality away from direct obstetric causes (due to the process of birth itself) towards indirect causes (due to deaths from other causes which are aggravated by the physiological effects of pregnancy or childbirth) (Souza et al., 2014). Like the demographic and epidemiological transition models, the obstetric transition is formed of several stages, and it is only in the fourth stage, when maternal mortality ratios have fallen to less than 50 maternal deaths per 10,000 live births, that indirect causes dominate. At this stage, indirect causes will be particularly due to non-communicable diseases. This conceptualization of the transition implicitly assumes, however, that the available statistics on maternal mortality over time and across space are complete and comparable. This assumption can be questioned as maternal mortality statistics are known to be subject to considerable underestimation, even in countries with complete vital registration systems and procedures in place to ensure high quality recording (Salanave et al., 1999, Gissler et al., 2004, Knight et al., 2016). Underestimation may be even more extreme in places without complete registration, but the sorts of double checks and detailed enquiries which can measure underestimation are often lacking (Songane et al., 2002, Ronsmans and Graham, 2006'). Data collection, recording and coding practices are credited with variation in the degree of underestimation, but studies using recent data which have attempted to evaluate the effect of different ways of measuring maternal mortality on underestimation have been hindered by the fact that ‘the different definitions invariably group countries according to their development status’ (Betrán et al., 2005) and thus it is difficult to disentangle the degree of underreporting from real differences in maternal mortality. In a comparative historic context, different classification systems, including successive revisions of the International Classification of Diseases, have treated certain types of maternal death very differently, making comparisons between countries and over time problematic.

This paper examines the effect of variable reporting and coding practices on the measurement of maternal mortality in late nineteenth century Scotland, using recorded causes of death and women who died within six weeks of childbirth. This relatively high mortality setting provides data to compare different ways of measuring maternal mortality and to contrast urban and rural settings with different certification practices. We find that underreporting was most significant for indirect causes, and that indirect causes accounted for a high proportion of maternal mortality where the infectious disease load was high. However, distinguishing between indirect and direct maternal mortality can be problematic even where cause of death reporting appears accurate. Paradoxically, underreporting of maternal deaths was higher in urban areas where deaths were routinely certified by doctors, and we argue that where there are significant differences in medical provision and who reports a death, differences in maternal mortality may reflect certification practices as much as true differences. Better health services might therefore produce an illusion of low maternal mortality, rather than actually producing better maternal outcomes. We end the paper with reflections on the interpretation of maternal mortality statistics and implications for the concept of the obstetric transition.

## 2 Levels, trends and measurement of maternal mortality

Maternal death is the death of a woman while pregnant or within a certain number of days of the termination of a pregnancy from any cause related to or aggravated by the pregnancy or its management but not from accidental or incidental causes. It can be divided into direct mortality, which arises directly from the pregnancy (for example post-partum haemorrhage, puerperal eclampsia, and puerperal fever), and indirect mortality, which is from non-pregnancy related causes which become aggravated by the pregnancy (for example an expectant mother may be more likely to catch and die from influenza). Maternal mortality can be estimated using a variety of different methods and definitions (Betrán et al. 2005). Places with reliable registration systems generally use cause of death information, while places and eras without good registration systems are more likely to rely on sample populations to identify deaths within a certain time since the birth of a child.

Studies of historic maternal mortality in the UK can be divided into two periods dominated by these different recording methods based on type of source (Galley and Reid, 2014). The earliest estimates of maternal mortality in the UK refer to the late sixteenth century and are based on the numbers of women dying within 60 days of a live birth, identified by linking the births and deaths within parish registers (Dobbie, 1982, Schofield, 1986, Wrigley et al., 1997). Despite being based on samples of places and subject to adjustments for background mortality and women dying without having delivered a live birth, these estimates generally match well with contemporary estimates obtained from reports of causes of death in the form of the London Bills of Mortality (Woods and Galley, 2014, p.21-3). Estimates from parish registers for the early nineteenth century also match well with those obtained from causes of death on the newly introduced death registers in the mid-nineteenth century lending confidence to the estimates (Woods and Galley, 2014, p.21-3). In the UK maternal mortality peaked in the late seventeenth century, at a time when all-cause mortality was also high, and declined gradually from then until the early nineteenth century when it stagnated at levels of about 55 maternal deaths per 10,000 live births until major declines in the 1930s and 40s.

The literature on maternal mortality in historic Europe and North America concentrates on midwifery and obstetric care, maternal nutrition and disease loads as factors behind differing levels and trends. The development of sulphonamides and antibiotics has been credited with the rapid decline in maternal mortality which started in the 1930s (Loudon, 1987, Loudon, 1992), but improvements in midwifery and obstetric care have also been linked to better maternal survival in parts of nineteenth and twentieth century Europe (Loudon, 1997, Högberg, 2004, Curtis, 2005, De Brouwere, 2007). Woods and Shelton (1997) suggested that registration practices might affect geographical patterns and it is this aspect of maternal mortality, the measurement and recording of it, on which this paper concentrates.

### 2.1 Measuring maternal mortality from causes of death

Although the identification of women dying in childbirth using cause of death might seem straightforward, it is in fact fraught with difficulty. Even in countries where most, if not all, deaths are certified by a medical professional, it is estimated that between 25 and 70 percent of maternal deaths are misreported (British Medical Bulletin 2003, p.2). The problem may have been much greater during the nineteenth century when the accuracy of cause of death recording in general was hampered by the pitfalls of rapid advances in medical knowledge and ideas about disease causation, changing nosologies, a lack of clarity in cause of death reporting, and relatively high levels of deaths which were not medically certified (Alter and Carmichael, 1996, Alter and Carmichael, 1998, Williams, 1996, Risse, 1997, Alter and Carmichael, 1999, Kunitz, 1999, Anderton and Leonard, 2004, Reid and Garrett, 2012, Reid et al., 2015). There were additional problems with the recording of maternal death, however. It was felt that doctors might be averse to confessing that they had attended a maternal death - because it might suggest incompetence to potential clients - and that they would be tempted to 'hide' the death by ascribing a different cause - usually one which might be at least partially correct but omitted to mention the crucial detail of a recent childbirth. Examples might include ascribing a death to 'haemorrhage' or 'peritonitis', rather than 'post-natal haemorrhage' or 'puerperal peritonitis'. In 1881 the Registrar General of England and Wales started to conduct special follow-up investigations, whereby individual doctors registering deaths were contacted and asked to provide more information about particular instances. These enquiries revealed a considerable number of maternal deaths which had been originally undetected, although it is likely that not all under-reporting was exposed (Loudon, 1992). Even when doctors did mention childbirth on the death certificates, these were not always allocated to maternal death categories in local and national statistics. Deaths were supposed to be coded to the antecedent cause (e.g., in the case of pneumonia following measles, measles is the antecedent cause as pneumonia is a complication - a direct consequence of having contracted measles), but researchers have noted that this was not always done systematically or accurately (Williams, 1996, Kippen, 2005, Reid et al., 2015).

Indirect causes of death are particularly problematic. This category contains deaths precipitated by a non-childbirth related cause which might not have occurred at all had the woman not been pregnant or recently delivered, their risk being increased by a pregnancy-related immunological transformation which increases vulnerability to a range of infectious diseases (Weinberg, 1984, Schofield, 1986, Reid, 2005, Racicot et al., 2014). Such deaths include causes such as ‘heart disease following childbirth’ or ‘tuberculosis exacerbated by childbirth’. The international cause of death coding system used today, ICD10, contains codes for such deaths and many death certificates now have a box to indicate if the deceased was pregnant or recently delivered. However, this was not the case for early versions of the ICD and its country-specific precursors, or for historic death certificates. In the past, doctors concentrating on the immediate cause of death and not realizing the contribution of the parturient state, might have failed to mention pregnancy or childbirth.

Again, coding issues may be particularly important for indirect causes. Although today such deaths are routinely included in maternal mortality, in the mid-nineteenth century opinion varied as to whether they should be counted in maternal deaths. The argument for including them was based on the fact that some women would not have died had they not been pregnant. The argument against was based on the fact that many would have died anyway, and that indirect mortality therefore really reflects levels of background mortality among non-pregnant women in the child-bearing ages, differences in which could then disguise levels and trends in ‘true’ maternal mortality. In late nineteenth century Scotland, maternal deaths were supposed to be included in maternal mortality, but there was no sub-category to enable them to be separately assessed using official statistics.

It is noteworthy that the accuracy of reporting by doctors and of coding by clerks were still major causes of underreporting of maternal mortality in late twentieth-century Europe and North America. Studies have found that very recently the underreporting of maternal mortality was considerably greater for indirect than for direct maternal deaths, and have speculated that different levels of under-recording in different European countries in the late twentieth century might be connected to differential certification and coding practices (Karimian-Teherani et al., 2002; Turner et al., 2002). Deaths due to maternal mortality but not described or coded as such have been termed ‘hidden’ maternal deaths (Loudon, 1992).

### 2.2 Measuring maternal mortality using time since birth

**Fig. 1:**
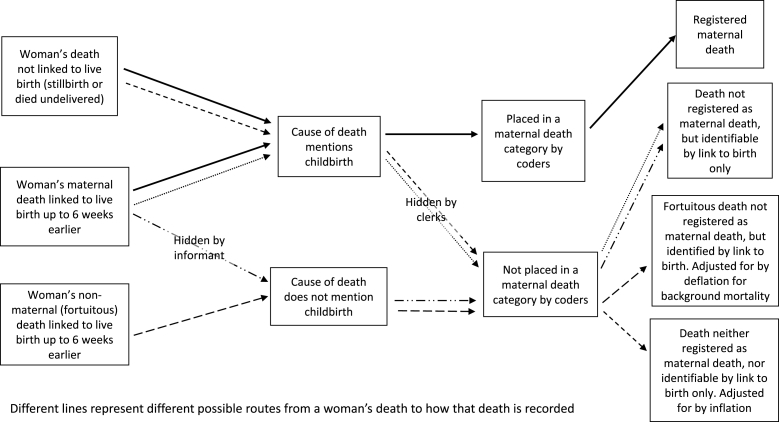
Identifying and recording maternal deaths.

One way of identifying hidden maternal deaths as described above is to use an approach to measuring maternal mortality which is usually employed where cause of death is not available, but life histories or longitudinal data are. Life histories enable the identification of women dying within a certain time (usually six weeks) after the birth of a child. Maternal mortality defined in this way will include a different set of women to those identifiable by cause of death: all direct and indirect deaths to women who produced a live birth will be included, as will any deaths wholly unrelated to the pregnancy, termed ‘fortuitous’ deaths. However, this method is not necessarily a more comprehensive method than examination of cause of death. As well as including fortuitous deaths which are not really maternal, it often excludes deaths to women who deliver a stillbirth or who die undelivered. These constitute another category of ‘hidden’ deaths (Schofield, 1986). For studies such as the current one, which rely on linkage to birth registers, this is a major issue because stillbirths were not recorded in British civil registers until well into the twentieth century (1927 for England and Wales and 1937 for Scotland). Some estimates suggest that 55 to 60 percent of maternal deaths are associated with a stillbirth, therefore this method can also substantially underestimate maternal mortality (Kippen, 2005). Some studies adjust for both background (including fortuitous) mortality and women dying undelivered (Schofield, 1986), but the extent to which we should expect the proportion of women dying undelivered to be constant over time is unclear. Fig. 1 depicts the steps through which a maternal death could be registered as maternal or hidden in various ways.

### 2.3 Measuring maternal mortality using different methods

Studies of underreporting of maternal mortality in high income, low mortality countries generally use a variety of methods including death certificates, data linkage, and direct enquiries to doctors to establish the degree of under-reporting and to improve the completeness of recording (Deneux-Tharaux et al., 2005, Horon, 2005, Nair et al., 2017). However, these tools are not always available in high mortality countries where death certification is frequently less complete and the sources and methods used vary systematically with levels of mortality (Betrán et al., 2005). It has therefore been very difficult to establish the effect of variable certification practices in high mortality settings. Historical data provide analogous possibilities using data linkage, but the coincidence of cause of death data and linked registers is not often available. Pantin (1996) compared officially recorded numbers of maternal deaths with original death registers for the Isle of Man (1882–1961) and found that coding and classification mistakes resulted in underestimation of maternal mortality in the official record. Kippen's study of the death registers of Tasmania found that the number of reported maternal deaths in the statistical reports could be increased by 35 percent when deaths were more accurately attributed to the right classification (Kippen, 2005). She then identified women who had recently given birth by linkage to the birth registers and this yielded a further clutch of maternal deaths. These additional deaths meant that the new total of maternal deaths was increased by a further 31 percent. Finally, she adjusted for the fact that some women whose deaths were not attributed to a maternal cause will have died in childbirth without delivering a live-born infant, by assuming that the proportion of maternal deaths not associated with a live birth was the same among those without a maternal cause as among those with a maternal cause. This resulted in a further 21 percent increase. Her final total of maternal deaths for Tasmania, 1838–1899, was more than double that reported in the official statistics.

This paper takes a similar approach to that of Kippen, focusing on two contrasting places in Scotland: the Hebridean Isle of Skye and the lowland town of Kilmarnock, over the course of the second half of the nineteenth century (1861–1901). As well as assessing the extent of underestimation due to misclassification, misrecording and dying without delivering a live infant, we examine the roles of medical certification of death and of background mortality in assessing the contrasting experiences of the urban and rural settings.

## 3 Materials and methods

### 3.1 The data

This paper uses the civil registers of birth, marriage and death between 1861 and 1901 for the town of Kilmarnock and the island of Skye, to which special access was granted by the Registrar General for Scotland. These data, and the entire populations of Skye and Kilmarnock in the five decennial censuses (freely available) between 1861 and 1901 inclusive, were transcribed into a machine-readable database. The events to individuals in each source were linked to each other using a semi-automated process. Links between dying women and the births they had in the preceding years can be made with confidence due to the wealth of detail in Scottish registration certificates, which record mother's maiden name on birth certificates and deceased's maiden name on death certificates (see Reid et al., 2006 for more detail on this dataset). The quality of civil registration in nineteenth century Scotland is reckoned to be very high, with almost complete coverage of death certification from its introduction in 1855. The original individual registrations of death provide the exact cause as assigned by the doctor or other informant, and the length of last illness. They also give the date of death which can be combined with date of birth on birth certificates to calculate time since the birth of a child. Death certificates further state whether a death has been medically certified or not and if so, provide the name of the certifying doctor. These doctors have been identified in the Medical Registers of the period, to ascertain, among other information, details of their qualifications, university cohort and publications.

Kilmarnock and Skye were very different places: Kilmarnock was a growing industrial town in the lowlands of Scotland, whose population increased from c.23,500 in 1861 to c.33,100 in 1901. It had a range of industries including textile manufacture, railway coach building, tobacco spinning and mining. It was easily accessible from Glasgow and was home to around five doctors per 10,000 inhabitants over the forty years between 1861 and 1901. The vast majority of the roughly 23,700 deaths occurring between 1861 and 1901 were certified by a doctor. This does not mean that a doctor attended to all patients in their final illness, as some doctors wrote death certificates having only seen the deceased after death. However, it does suggest that medical services were available, at least in cases of dire need.

The Isle of Skye in the Inner Hebrides also had around five doctors per 10,000 people over this period. However, the low and declining population density (the population declined from 19,600 in 1861 to 14,600 in 1901) and the physical characteristics of the island, whose ragged coastline and mountainous interior still lead to long journey times today, meant that doctors were physically much less accessible than in Kilmarnock. This is demonstrated by the fact that less than half of the c.12,600 deaths occurring in our study period were certified by a doctor. For the rest, no medical attendant was available either during the final illness or shortly after the death.

### 3.2 Calculating maternal mortality for Kilmarnock & Skye

In order to identify and categorise maternal deaths we took the following steps (described in more detail in online Appendix 1):

1) We identified all maternal deaths using the cause of death information on the death certificates.
2) We then identified additional deaths to women within six weeks of the birth of a child by nominal linkage from women's death certificates to the births of their children.
3) We used maternal deaths for Kilmarnock to work out how nineteenth century coding clerks would have categorised different causes by seeing what ‘rules’ would produce the same annual numbers of deaths from maternal mortality and puerperal fever as were published in the Registrar General (RG)’s reports. It was necessary to take this indirect route as no documents giving instructions to coders have survived.
4) We coded all maternal deaths identified either by cause or by time since birth using our inferred RG's coding rules and our own classification which pays attention to secondary causes and recent childbirths even if they were not mentioned on the death certificate. This new classification also separates out deaths from toxaemia and haemorrhage as well as those from puerperal fever, other direct maternal deaths, and indirect deaths.
5) We made an adjustment for women who died without delivering a live-born infant, by assuming the ratio of those without a maternal cause to those with a maternal cause was the same among those for whom a live birth cannot be identified as for those for whom a live birth can be identified. We provide two estimates for this, the higher of the two uses separate ratios for direct and indirect maternal causes to calculate the additional hidden deaths and then adds these together. It is higher because indirect maternal causes were more likely to have not been given a maternal cause of death on the death certificate. We also provide the estimate from the combined rates because we are not wholly confident that direct and indirect maternal mortality are accurately distinguished from one another. These two estimates can be regarded as the upper and lower bounds of a range.
6) We adjusted for background mortality by subtracting the expected number of deaths in the six weeks following childbirth among women who had given birth, based on non-maternal deaths to women of childbearing age in each place.

## 4 Results

The results of these procedures are shown in Table 1, which shows both numbers of maternal deaths and maternal mortality ratios (the number of maternal deaths per 10,000 live births) for direct causes, indirect causes and both together. The following points can be drawn from the table:

1) Comparison of the first row (contemporary classification) and the subsequent rows shows that underestimation of maternal mortality in nineteenth century Scotland was severe. Nineteenth century categorizations resulted in rates which were underestimated by at least one third on Skye and over a half in Kilmarnock.
2) Underestimation was due to a combination of mis-categorisation of recorded causes of death, but especially to the failure to provide a maternal cause of death on death certificates. If accurate causes had been given, there would not be a need to inflate for women who died without delivering a live infant, so this inflation can also be attributed to the failure to provide a maternal cause.
3) Although direct maternal deaths were sometimes mis-categorised or given a misleading cause, the problem was much greater for indirect maternal deaths.
4) Underestimation was far greater in the urban area, Kilmarnock, than on the rural Isle of Skye.
5) Therefore, although the ‘official’ rates (contemporary classification) suggest that the rural area had higher maternal mortality, in fact rates were higher in the urban area, particularly due to the contribution of indirect causes.

**Table 1:** Calculation of maternal mortality ratios, Kilmarnock and Skye, 1861–1901.

	**Kilmarnock**					
	direct maternal mortality		indirect maternal mortality		direct and indirect maternal mortality	
	deaths	MMR	deaths	MMR	deaths	MMR
contemporary classification	186	49	18	5	204	53
re-classification	200	52	40	10	240	63
including additional deaths within 6 weeks of birth	260	68	164	43	424	111
adjusting for those not delivering a live infant	276	72	208–253	55–66	484–529	127–139
adjusting for background mortality	276	72	166–211	44–55	442–487	116–128
	**Skye**					
	direct maternal mortality		indirect maternal mortality		direct and indirect maternal mortality	
	deaths	MMR	deaths	MMR	deaths	MMR
contemporary classification	138	78	1	1	139	79
re-classification	140	79	8	5	148	84
including additional deaths within 6 weeks of birth	147	83	33	19	180	102
adjusting for those not delivering a live infant	156	89	62–73	35–41	218–229	124–130
adjusting for background mortality	156	89	50–61	28–35	206–217	117–123

Notes: Maternal mortality ratios (MMR) are the number of maternal deaths per 10,000 live births. Ranges for adjusted estimates use different adjustment factors for direct and indirect mortality.

We argue in the following sections that the urban-rural differences in underestimation are due to the proportion of deaths where the cause was suggested by a doctor rather than a lay person, and differences in the causes offered by lay people as opposed to doctors. Differences in indirect mortality can be partly attributed to levels of background mortality, but there may also be difficulties in accurately distinguishing direct and indirect causes.

## 5 Medical provision and the certification of maternal death in town and country

Although Kilmarnock and Skye had similar numbers of doctors per head of population, the large area and sparse population on Skye meant that those doctors were in practice not accessible and that a large proportion of deaths were not medically certified, including 45 percent of maternal deaths. In contrast only one maternal death in Kilmarnock was not medically certified. This presents something of a paradox, as it might be expected that doctors would offer more accurate causes of death, but the problem of misleading causes of death was in fact more prominent in the town.

**Fig. 2:**
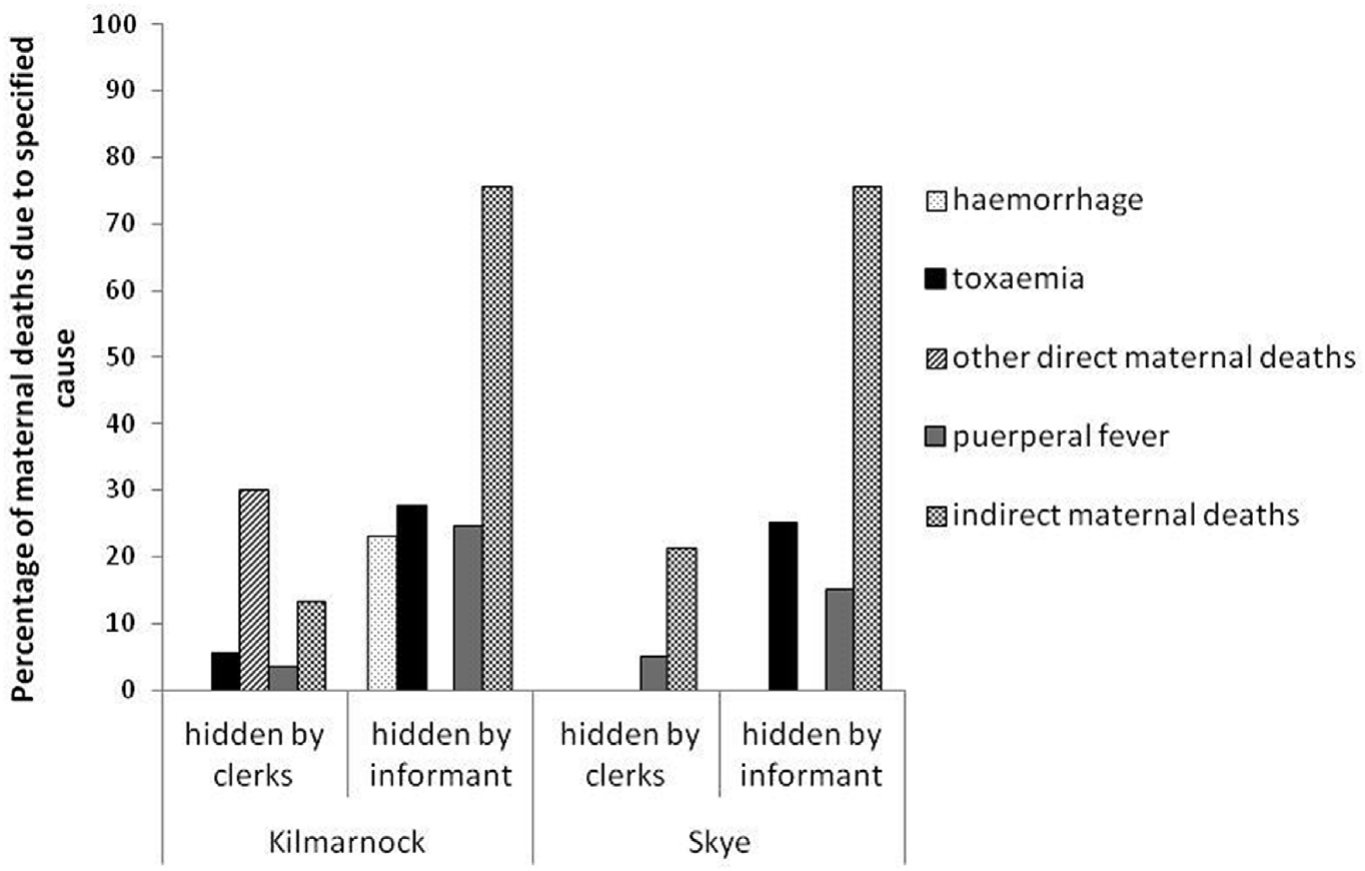
Percentages of maternal deaths hidden by clerks and informants, by cause of death and place, Kilmarnock and Skye 1861–1901. Notes: Some cause of death categories have small numbers so percentages should be treated with caution. This is particularly the case for toxaemia and haemorrhage on Skye (4 and 17 deaths respectively).

It has been suggested that doctors might try to deliberately ‘hide’ maternal deaths to protect their professional reputations. If this was so it might be particularly pertinent for cases of puerperal fever, in which the doctor's role in transferring the infection to the patient was becoming known. However, there is little support for this in these data. Fig. 2 shows that deaths that we can identify as puerperal fever were not significantly more likely to have been given a misleading cause by doctors (‘hidden by informant’) than other specific forms of direct maternal mortality. Moreover, some doctors accurately certified all the puerperal fever deaths that we can also identify (see online Appendix 2 for more information about the certification practices of individual doctors). It is more likely that thoughtlessness was at the root of the failure of the Kilmarnock doctors to certify maternal deaths in an informative way. This is supported by the case of one particular doctor, John Thomson, who admitted in articles published in the Glasgow Medical Journal to average rates of maternal mortality and puerperal fever, but whose maternal deaths in the dataset were recorded as ‘peritonitis’, ‘fever’ and ‘repeated haemorrhage’ (Thomson, 1855, Thomson, 1864. See Appendix 2 for more details). It is possible that doctors were trying to provide medically correct causes, but did not realize that in the absence of knowledge about a recent birth, these could not be allocated to maternal mortality.

**Fig. 3:**
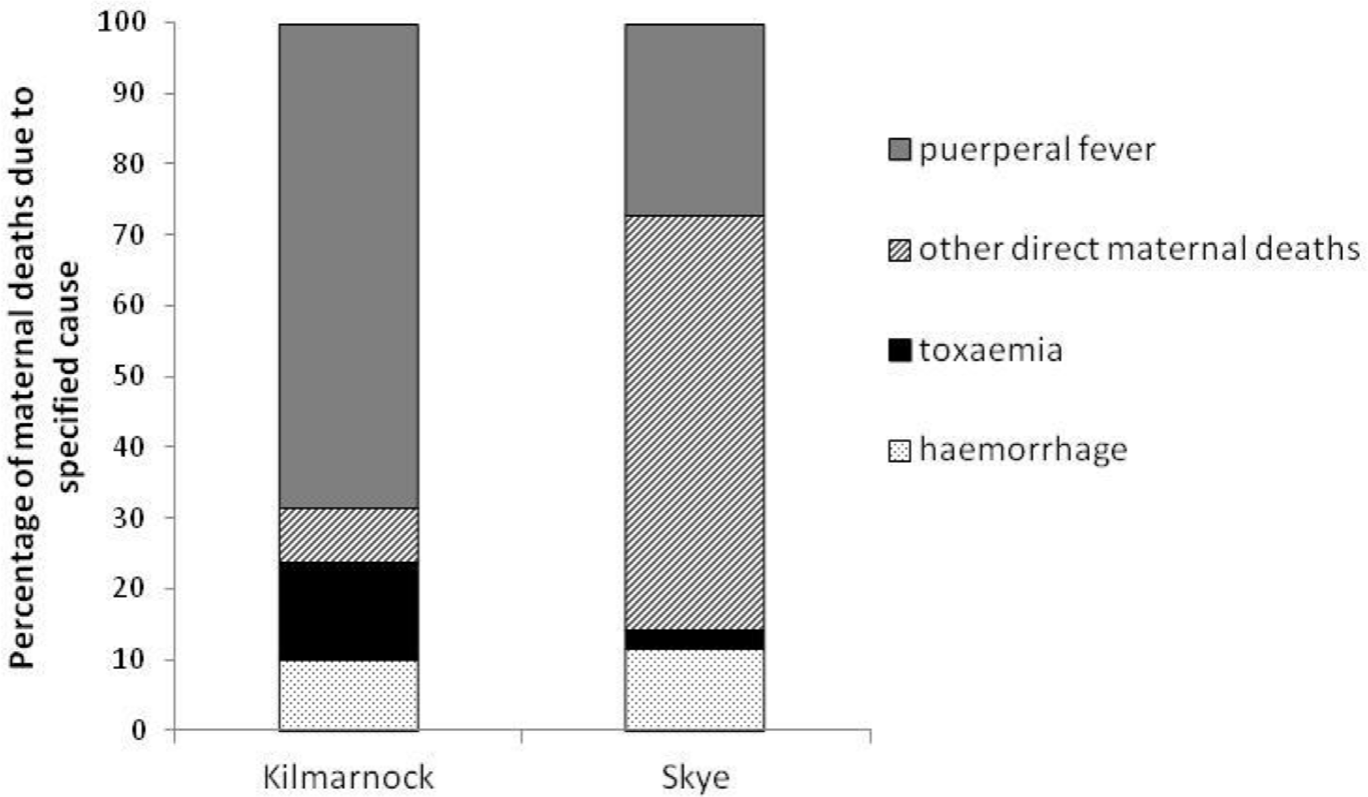
Distribution of direct maternal deaths by cause, Kilmarnock and Skye 1861–1901. Notes: Total numbers of direct maternal deaths in Kilmarnock and Skye are 260 and 147 respectively.

**Fig. 4:**
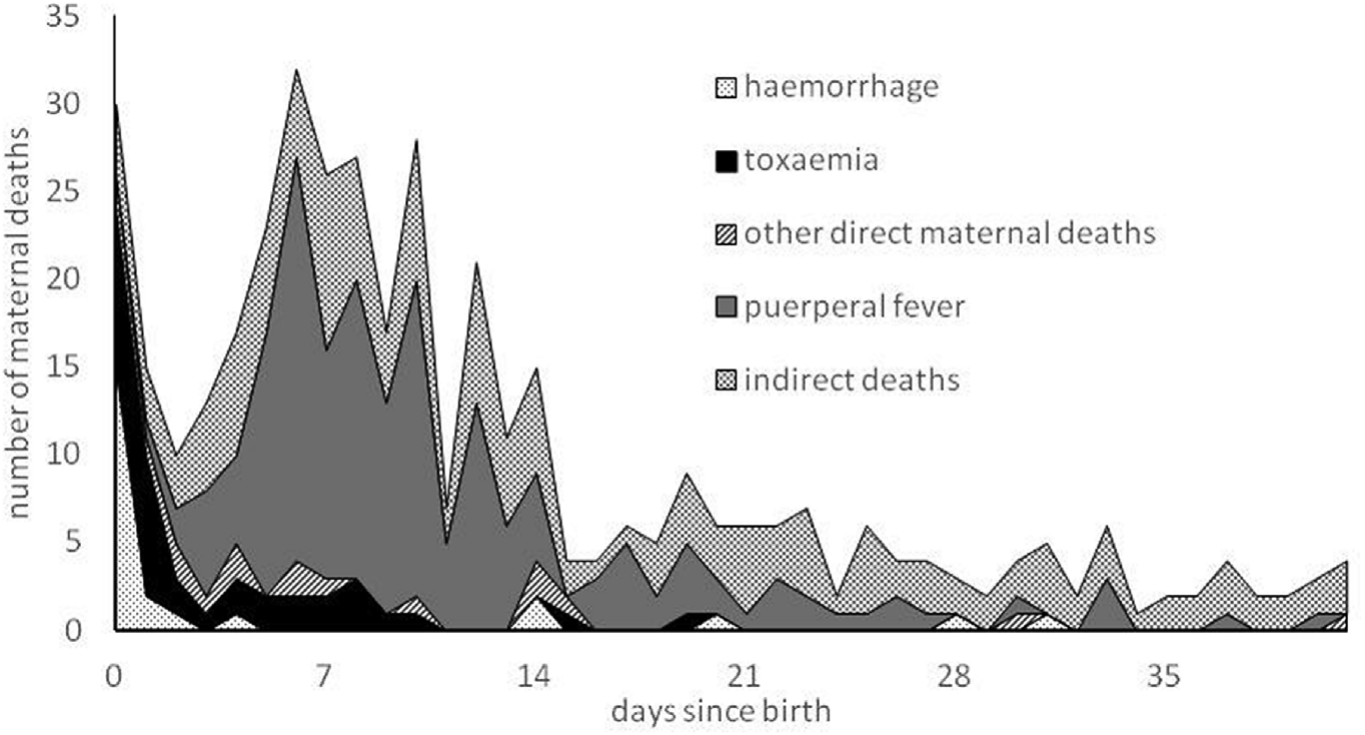
The number of post-partum maternal deaths by newly assigned cause and by interval between birth of child and death of mother, Kilmarnock 1861–1901. Notes: 43 deaths unlinked to a live birth have used duration of last illness in place of days since birth of child, and a further 18, unlinked to a live birth and where duration of last illness was not given, are excluded. Very similar patterns result when only deaths linked to the birth of a child are used.

**Fig. 5:**
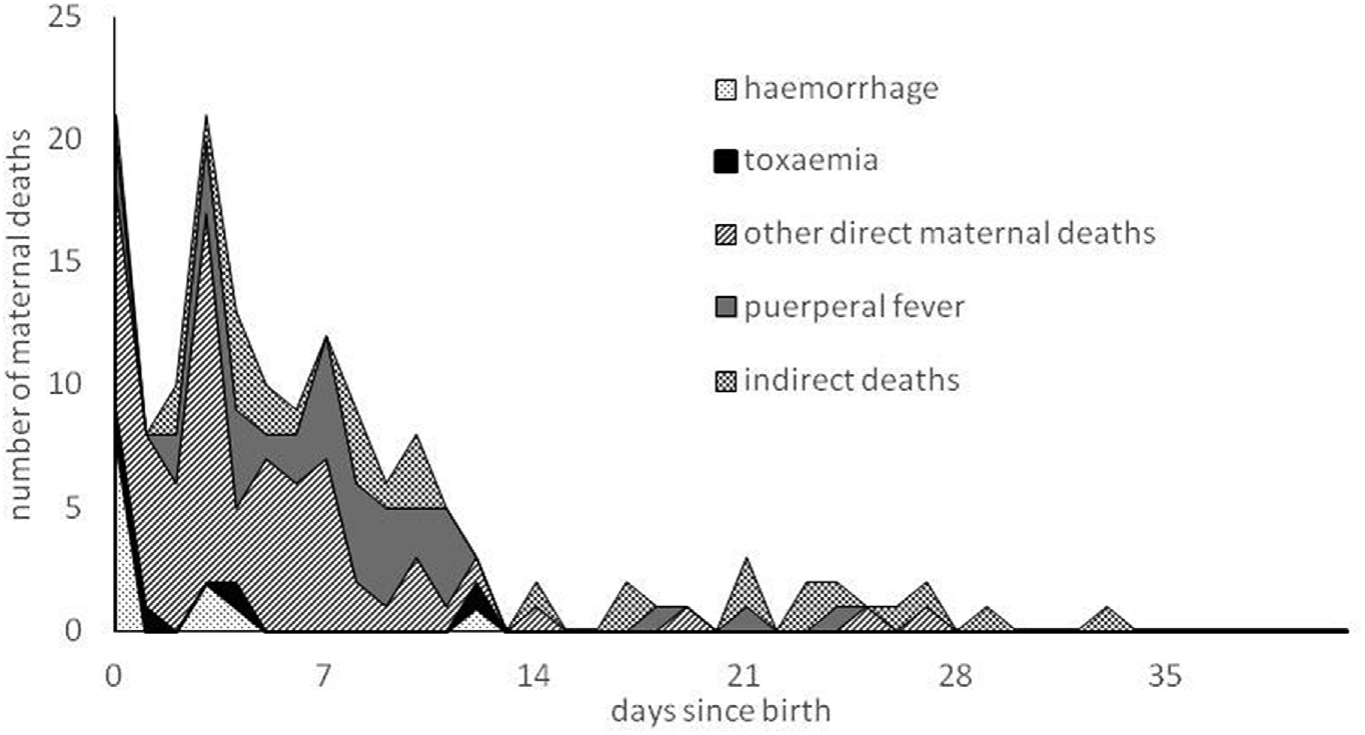
The number of post-partum maternal deaths by newly assigned cause and by interval between birth of child and death of mother, Skye 1861–1901. Notes: 56 deaths unlinked to a live birth have used duration of last illness in place of days since birth of child, and a further 23, unlinked to a live birth and where duration of last illness was not given, are excluded. Very similar patterns result when only deaths linked to the birth of a child are used.

Fig. 3 shows the distribution of direct maternal deaths over four broad causal groups – three specific categories (puerperal fever, haemorrhage, and toxaemia) and one residual category (other direct maternal deaths). The difference in the distributions between Kilmarnock and Skye is striking: in Kilmarnock, the majority (92 percent) of direct deaths were attributed to either puerperal fever, haemorrhage or toxaemia, but for Skye this is was the case for less than half of direct deaths. This is because the majority of maternal causes of death on Skye were simply written as 'childbirth' with no further details, but it is likely that many of these were actually due to a more specific cause. Comparison of the distributions of the different types of maternal death by time since birth, shown in Fig. 4, Fig. 5 for Kilmarnock and Skye respectively, confirms this.

It is well established that different causes have different patterns of mortality by time since birth, with deaths due to post-partum haemorrhage usually occurring on the day of birth or that following, toxaemia mortality occurring within the first week of life, but puerperal fever usually claiming victims between four and fourteen days after birth (Loudon, 1992). For Kilmarnock, it is clear that the different causes conform to the expected patterns, suggesting that our newly assigned causes are generally reliable. However, it is interesting that they do not conform to the proportions that Loudon (1992, p.3) states were generally invariant until the mid-1930s. His range of sources indicated that puerperal fever generally accounted for 33 to 50 percent of maternal deaths, toxaemia for about 20 percent and haemorrhage for 15 to 20 percent, irrespective of the level of maternal mortality. In late nineteenth century, Kilmarnock puerperal fever appears to have contributed a higher proportion (around two thirds) of direct maternal deaths, with haemorrhage and toxaemia only reaching 10 and 13 percent respectively (see Fig. 3). These percentages are very similar if we only consider deaths which would have been placed in maternal death categories at the time. The discrepancy may be due to the composition of other direct maternal deaths and of indirect deaths. The former includes some causes such as obstructed labour but are mostly ill-defined causes which should probably belong in one of the more tightly defined categories. In addition, some of the indirect deaths might actually have been produced by a direct maternal cause.

**Fig. 6:**
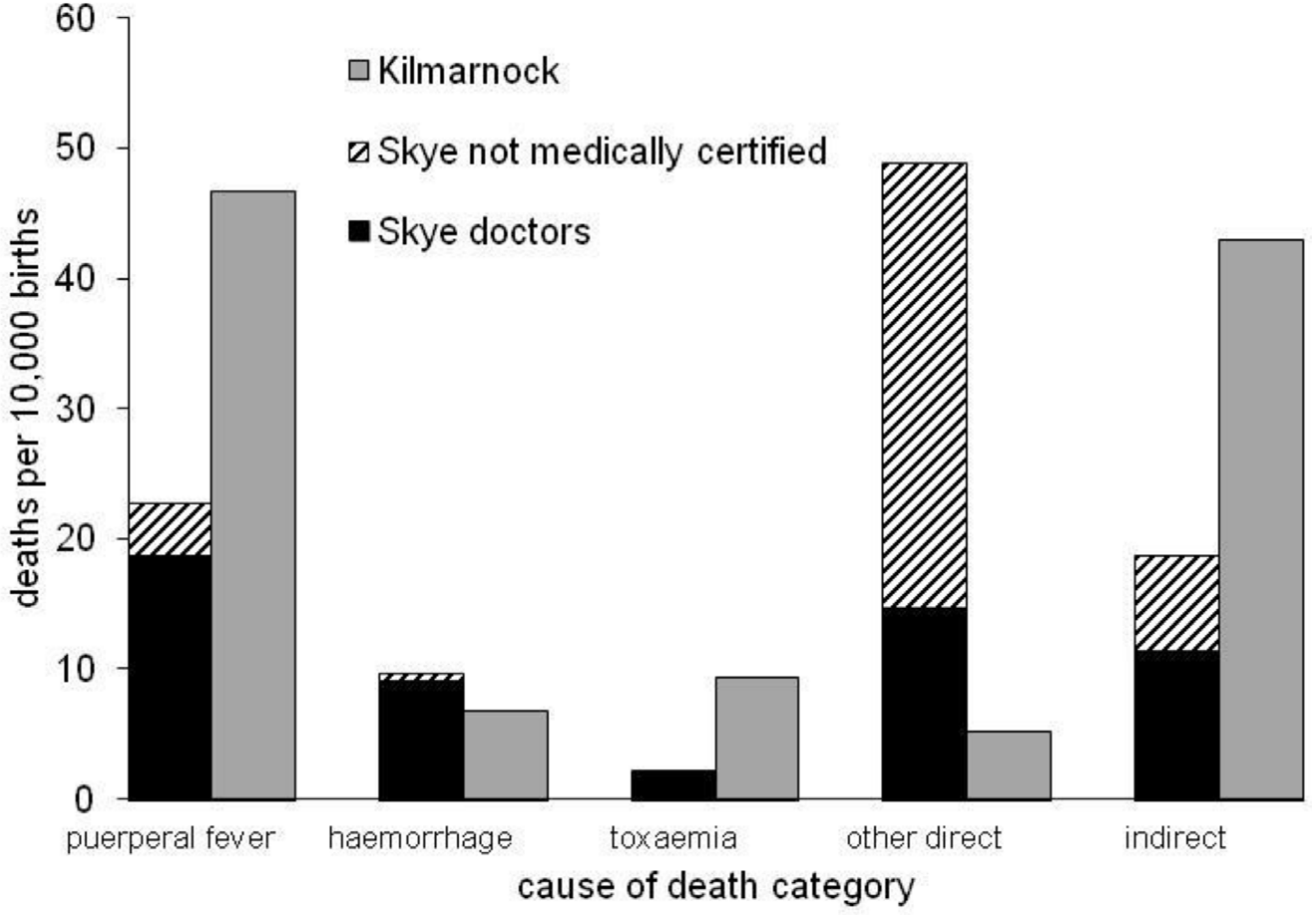
Maternal deaths per 10,000 births, for different causal categories: comparison between Skye doctors, Skye uncertified and Kilmarnock doctors.

Fig. 5 shows the distributions of causal groups by time since birth on Skye, and while there are reassuring similarities with Kilmarnock, there are also notable differences. The pattern of haemorrhage deaths is as expected, with the majority occurring on the day of birth. However, there are fewer than expected toxaemia deaths soon after birth, as well as more 'other maternal deaths'. We attribute this to low levels of medical certification of death which we explore in more detail in Fig. 6 which shows the number of maternal deaths from particular causes, distinguishing between those suggested by a doctor and by an informant, per 10,000 births on Skye over the period. Equivalent figures for Kilmarnock are shown for comparison.

Almost all deaths recorded as being from one of the specific maternal causes (puerperal fever, haemorrhage, and toxaemia) were reported by a doctor on Skye. It is likely to have been clear to lay informants (usually a close relative of the deceased) that childbirth was implicated in the death, but they did not have the vocabulary to identify a specific cause of death so simply reported ‘childbirth’ as the cause. Many of the 'other direct maternal deaths' may therefore belong in the other categories, perhaps even indirect deaths. However, doctors on Skye were also more likely than doctors in Kilmarnock to use non-specific childbirth terms, probably because they may have only seen the patient after her death. In these cases they would have relied on reports of family members who, on being questioned by the doctor, are likely to have been able to identify large quantities of blood loss enabling the doctor to write haemorrhage on the certificate, but were less proficient at identifying or recalling symptoms of toxaemia or puerperal fever. It is also possible that the risk of puerperal fever was lower on the island, with fewer incidents of septicaemia from industrial accidents and less contact with doctors to spread the infection.

It is also interesting to compare the risks of death from direct and indirect maternal mortality in the two places. There are convincing reasons for the risk of direct maternal mortality to be higher on Skye, such as fewer midwives to attend childbirth and a smaller chance that a doctor could arrive in time if there was a problem (see D’Ambruoso et al.(2010) for evidence that distance and transport availability contributed to maternal mortality in rural Burkina Faso and Indonesia). There are even more plausible reasons for the risk of indirect mortality to be lower, in that Skye had a significantly more benign disease environment than Kilmarnock, reducing incidence of the conditions implicated in indirect mortality. Background mortality among women of childbearing age on Skye, calculated by taking the number of deaths to women aged 15–44, subtracting maternal deaths and dividing by the number of women, was 58 deaths per 10,000 women, compared to 96 in Kilmarnock. On the other hand, there is also evidence that doctors did not always improve the likelihood of maternal survival: they were more prone to interfere unnecessarily during a birth, risking inflicting damage or introducing infection (Reid, 2012). It is therefore difficult to know whether the balance between direct and indirect mortality was real or a result of reporting variations: next of kin on Skye may have reported childbirth as the cause of death even if the woman died from a different disease. If we assume that indirect maternal mortality varies in relation to background mortality (Boerma, 1987), an assumption supported by evidence that indirect mortality tends to rise during epidemics such as the 1918 influenza epidemic, malaria and HIV/AIDS (Cross et al., 2010), we could expect indirect mortality on Skye to be about 40 percent lower than that in Kilmarnock. Causes of death and additional cases suggest it is 56 percent lower, indicating that there is likely to be some fuzziness between direct and indirect mortality.

It seems plausible from this analysis that where cause of death is relied upon to generate maternal mortality statistics, registration by doctors does not always produce an accurate picture. The division between direct and indirect maternal deaths was not well defined in late nineteenth century Scotland, and ignoring indirect deaths may lead to either overestimation of maternal mortality in rural areas or underestimation of it in urban areas. Comparable estimates should include both direct and indirect causes if possible.

## 6 Conclusions

This paper has examined the reporting of maternal mortality in nineteenth century Scotland, contrasting an urban and a rural setting with different certification of death practices linked to access to doctors. We have found that doctors often ‘hid’ maternal deaths, probably through a desire to be more precise. Therefore, it is possible that increasing medical certification of death and increasing attendance of doctors at births might create a spurious decline in reported maternal mortality, or disguise an increase. Doctors in Kilmarnock may have been more likely to have hidden deaths as indirect causes, but it is also likely that many indirect maternal deaths on Skye were recorded simply as 'childbirth', blurring the distinction between direct and indirect maternal mortality. Comparisons of overall and particularly cause-specific maternal mortality between settings with varying medical attendance during illness or childbirth or varying medical certification of death are therefore likely to be affected by differential underreporting. Where there are significant differences in medical provision and who reports a death, differences in maternal mortality may reflect certification practices as much as true differences. Comparisons over times when medical provision is subject to change and between urban and rural areas are therefore particularly problematic. The implications for the course of maternal mortality levels, trends and patterns in the UK are that maternal mortality is likely to have been underestimated, particularly in urban areas and increasingly over the course of the late nineteenth century as the medical certification of death increased. Chamberlain's claim for England and Wales that ‘it was not until about 1870, when the registration of cause of death was made mandatory, that rates became reasonably accurate’ is unlikely to be defensible and it is plausible that rates derived from parish registers and in the early years of registration, when causes of death were more likely to have been reported by family members, were actually more accurate (Chamberlain, 2006, p.559). If the perverse influence of medical provision on underreporting is repeated in other settings, then using maternal mortality as an indicator of the coverage and quality of health services (Songane and Bergström, 2002) may be flawed. Better health services might produce an illusion of low maternal mortality, or served to exaggerate just how low the rates were, rather than actually bringing about better maternal outcomes.

We have also shown that indirect maternal mortality was much more likely to have been underreported than was direct maternal mortality when overall maternal mortality levels were relatively high, and that indirect mortality was strongly connected to general levels of background mortality. In Kilmarnock, almost half of all maternal deaths may have been due to indirect causes when maternal mortality was well over 100 per 10,000 live births. These observations prove problematic for the ‘obstetric transition model’ which states that as maternal mortality declines, there will be a shift in the balance of maternal mortality away from a predominance of direct obstetric causes and towards more indirect causes (Souza et al., 2014). In general, settings with high maternal mortality may be particularly vulnerable to the underreporting of indirect maternal mortality (Ronsmans and Graham, 2006, Nair et al., 2017) and the dominance of direct causes is cast into doubt if indirect causes are more likely to be underreported or to be attributed to direct causes because they were reported in vague terms, such as the one simple word: 'childbirth'.

Although we do not challenge the idea, inherent in the obstetric transition model, that indirect causes are likely to increase *as a proportion* of maternal mortality due to the better control of direct causes, we argue that it is likely that indirect causes have always been high. Under disease regimes with higher infectious disease loads, such deaths are likely to be dominated by infectious diseases. Denaux-Tharaux et al (2005) suggest that 'in areas where a large proportion of pregnancy-related deaths are indirect, pregnancy-related mortality may be more an indicator of the health status of reproductive aged women'. We argue that this is generally true of indirect maternal mortality at all times and that it is a fatal flaw of the obstetric transition model to imply that indirect maternal mortality might be low when the disease load in the environment is high. Models such as the epidemiological and obstetric transitions which rely on causes of death, particularly those vulnerable to problems with cause of death reporting and coding, should be treated with caution.

## Conflicts of interest

None.

## Footnotes

Appendix A Supplementary data related to this article can be found at https://doi.org/10.1016/j.socscimed.2018.01.028.
