# Supplementary material for: Medical provision and urban-rural differences in maternal mortality in late nineteenth century Scotland
Source: Soc Sci Med. 2018 Mar;201:35–43. doi: 10.1016/j.socscimed.2018.01.028 (PMC6565842; doi:10.1016/j.socscimed.2018.01.028)
Supplement: Online data [file mmc1.pdf]

Medical provision and urban-rural differences in maternal mortality in late nineteenth century Scotland

Alice Reid and Eilidh Garrett:

Social Science and Medicine 2018

Online Appendices

Appendix 1. Identifying and categorising maternal deaths

1) Identification of maternal deaths using the cause of death information on the death certificates:

We searched for anything mentioning childbirth, travail, metritis, metria, parturition, abortion, miscarriage, pregnancy; words such as puerperal, post-partum, and placenta, as well as other known conditions of childbirth such as eclampsia.

2) Identification of additional deaths to women within six weeks of the birth:

Death certificates give the deceased's forename and surname, maiden surname and, if they were married, the name of their spouse. Birth certificates give the name and surname of the father and name and maiden surname of the mother of the child. For each woman dying aged between 15 and 54 we searched for births in the year before her death, matching on her own and her husband's name (taking the absence of a husband in both sources as a match). In our analysis we considered deaths within six weeks of childbirth as possible maternal deaths. We calculated days since birth of child from dates of death of mother and birth of child.

3) Working out nineteenth century coding 'rules':

Kilmarnock was one of Scotland's 'larger towns' and consequently deaths for each causal group were published annually in the Registrar General for Scotland's detailed annual reports. Between 1

and 13 deaths were classed by the registrar general's office as due to 'childbirth' or 'metria' (puerperal fever) in each of the 40 years covered by the individual level data. On an annual basis it is therefore possible to take all individual death certificates where pregnancy or childbirth were mentioned or implied, and try to work out how they might have been assigned to a cause of death category by the registrar's clerks.

As an example, Table A.1 shows the maternal deaths for 1862, a year in which the Registrar General's reports returned three deaths as due to childbirth and none to metria. In this case we have assumed that the three deaths in which childbirth is clearly implicated in the first mentioned cause are those that contemporaries classified as childbirth. However we can see that there was one further death in which childbirth was mentioned as a secondary cause. Cases such as this over the entire data set indicate that for the most part (but not always) nineteenth century coding clerks ignored all causes apart from the first when classifying deaths. There were other cases (although not the one in this year) where they appear to have assumed that causes such as 'rupture of the womb' and 'inflammation of the uterus' were related to child birth, and so we have included four additional such deaths as maternal deaths even were a birth could not be found.

Table A.1: Examples of assigning cause of death groups to maternal deaths, Kilmarnock 1862.

days since birth of child	length of last illness	original cause of death	probable contemporary causal group	new causal group
0	few seconds	apoplexy	diseases of the brain	toxaemia
3	5 & 4 dys	diarrhoea, peritonitis	diarrhoeal diseases	puerperal fever
5	8 dys	bronchitis	respiratory diseases	indirect causes
6	10 dys	inflammation of uterus, puerperal fever	other reproductive diseases	puerperal fever
6	14 hrs	puerperal convulsions	child birth	toxaemia
8	7 dys	peritonitis	peritonitis	puerperal fever
9	2 yrs	fever	infectious diseases	puerperal fever
	14 dys	flooding after abortion	child birth	haemorrhage
19	3 wks	puerperal peritonitis	child birth	puerperal fever
38	18 mths	consumption	tuberculosis	indirect causes

4) Categorisation of maternal deaths:

We categorised all maternal deaths, whether identified by cause or by time since birth, using two different schemes: firstly the nineteenth century 'rules' as inferred in step 3), and secondly a more modern classification which pays attention to secondary causes and recent childbirths even if they were not mentioned on the death certificate. This second classification also separates out deaths from toxaemia and haemorrhage as well as those from puerperal fever, other direct maternal deaths, and indirect deaths.

In our assignment of new categories we made the following assumptions: any death (within 42 days of the birth of the last child) from an infectious disease, whether or not childbirth was mentioned, was classed as indirect maternal mortality. Any death within 42 days of the birth of a live child which was the result of convulsions, apoplexy or uraemia was assigned to toxaemia (eclampsia), and deaths from inflammation of the uterus and fever were assigned to puerperal fever. Other deaths within 42 days with specified causes or where childbirth was not mentioned were classed as indirect causes. Those where childbirth was mentioned and any other mentioned cause was vague or symptomatic were placed in ‘other direct maternal deaths’. On Skye there were a small number of deaths where the cause was stated to be 'unknown' and these were allocated to indirect maternal mortality. The fact that childbirth was not mentioned and that many causes were vague means that there is likely to be considerable uncertainty and overlap between ‘other direct maternal deaths’ and ‘indirect deaths’. Table A.1 also shows new maternal death causal groups for the Kilmarnock maternal deaths in 1862.

Tables A.2 and A.3 cross-classify, for Kilmarnock and Skye respectively, the probable contemporary causal group with the new causal group, the rows showing the category probably used by the Registrar General, and the columns showing the new classification. Most re-classified deaths fell into either puerperal fever or indirect causes, with several ‘fever’ deaths (under the contemporary category of infectious diseases) and a substantial number of ‘peritonitis’ deaths revealed to be maternal mortality deaths. However in Kilmarnock larger proportions of toxaemia/eclampsia deaths had been described as 'apoplexy' which was classed by contemporaries as a disease of the brain and is usually assumed to have referred to stroke. Indirect maternal deaths had the highest proportion of hidden deaths, mostly because doctors did not mention the woman's recent birth. These are drawn from a wide variety of other causes, from respiratory tuberculosis and other respiratory diseases, to digestive diseases and heart diseases.

Table A.2: Cross-classification of probable contemporary and new causal groups for maternal deaths and deaths within 6 weeks of childbirth, Kilmarnock 1861-1901.

probable contemporary cause	puerperal fever	haemorrhage	toxaemia	other direct maternal deaths	indirect maternal deaths	Total
child birth	28	20	22	14	18	**102**
metritis/puerperal fever	100		2			**102**
peritonitis	25					**25**
infectious diseases	4				10	**14**
diarrhoeal diseases	3	1			37	**41**
diseases of the heart					19	**19**
tuberculosis					32	**32**
respiratory diseases					28	**28**
diseases of the brain			8		4	**12**
joint diseases					4	**4**
kidney diseases			1		2	**3**
other reproductive diseases	8	3		4	1	**16**
other causes	10	2	3	2	9	**26**
**Total**	**178**	**26**	**36**	**20**	**164**	**424**
% of direct maternal deaths	68.46	10.00	13.85	7.69		
% 'hidden by registrar/clerks'	3.37	0.00	5.56	30.00	13.41	8.49
% 'hidden by doctor'	24.72	23.08	27.78	0.00	75.61	43.40

Notes: 'Hidden by registrars/clerks' indicates that the certificate mentioned childbirth as a secondary cause. 'Hidden by doctor' indicates that the cause did not mention or imply childbirth. Misclassifications between childbirth and puerperal fever are not counted as hidden.

Table A.3: Cross-classification of probable contemporary and new causal groups for maternal deaths and deaths within 6 weeks of childbirth, Skye 1861-1901.

probable contemporary cause	puerperal fever	haemorrhage	toxaemia	other direct maternal deaths	indirect maternal deaths	Total
child birth	5	17	3	86	1	**112**
metritis/puerperal fever	27					**27**
peritonitis	3					**3**
infectious diseases	4				5	**9**
diarrhoeal diseases					1	**1**
diseases of the heart					3	**3**
tuberculosis					2	**2**
respiratory diseases	1				11	**12**
diseases of the brain			1			**1**
joint diseases					2	**2**
kidney diseases						**0**
other reproductive diseases						**0**
not known					8	**8**
**Total**	**40**	**17**	**4**	**86**	**33**	**180**
% of direct maternal deaths	27.2	11.6	2.7	58.5		
% 'hidden by registrar/clerks'	5.0	0.0	0.0	0.0	21.2	**5.0**
% 'hidden by doctor'	15.0	0.0	25.0	0.0	75.8	**17.8**

Notes: 'Hidden by registrars/clerks' indicates that the certificate mentioned childbirth as a secondary cause. 'Hidden by doctor' indicates that the cause did not mention or imply childbirth. Misclassifications between childbirth and puerperal fever are not counted as hidden.

5) Adjustment for women who died without delivering a live-born infant:

We have inflated the number of maternal deaths using the assumption that the ratio of those without a maternal cause to those with a maternal cause was the same among those for whom a live birth cannot be identified as for those for whom a live birth can be identified. Table A.4 summarizes this process for Kilmarnock and Skye, using maternal mortality ratios, defined as the number of maternal deaths per 10,000 live births. The upper left ratio in each panel includes only deaths identified by both cause of death and time since birth, the upper right ratio uses those identified by the time since birth method only, and the lower left ratio uses those identified by the cause method only. The lower right ratio for each place includes two further estimates of maternal deaths hidden to both methods. These both assume the ratio of those without a maternal cause to those with a maternal cause was the same among those for whom a live birth cannot be identified as for those for whom a live birth can be identified. The second of these two estimates uses separate ratios for direct and indirect maternal causes to calculate the additional hidden deaths and then adds these together. It is higher because indirect maternal causes were more likely to have not been given a maternal cause of death on the death certificate. We also provide the estimate from the combined rates because we are not wholly confident that direct and indirect maternal mortality are accurately distinguished from one another. These two estimates can be regarded as a range.

Table A.4: Maternal mortality ratios, Kilmarnock and Skye 1861-1901

Kilmarnock

	cause identified as maternal	including those hidden to cause method
linked to live birth	47	95
including those hidden to birth method	63	*127-139*

Skye

	cause identified as maternal	including those hidden to cause method
linked to live birth	39	57
including those hidden to birth method	84	*124-130*

Notes: maternal mortality ratios are measured as the number of maternal deaths per 10,000 live births. No adjustment is made for background mortality in this table.

It is noteworthy that the adjustment for women dying without delivering a live birth is larger for Skye than for Kilmarnock. This might signify a higher rate of stillbirths or of women dying undelivered, but alternatively it might indicate some under-registration of live births. Such under-registration has been suggested as a reason for higher maternal mortality in rural areas (Woods and Shelton 1997). The link between under-reporting and higher maternal mortality is plausible in that generally reporting of deaths was more complete than reporting of births, and live births where no death was involved might have escaped certification. However it is unlikely that under-reporting of live births will have affected this ratio as it is implausible that family members registering a maternal death would not have registered the birth at the same time (cf Kippen 2005).

This difference in the proportion of deaths which cannot be linked to a live birth is worth some scrutiny. Kippen (2005) finds that among seven historic and contemporary studies, including her own, between 40 and 45 per cent of maternal deaths were not associated with a live birth. Ronsmans and Graham (2006) cite an even larger proportion (more than half) for Bangladesh. At 54 per cent (for those with maternal cause given) the Skye figure is fairly close, and deficiencies in death registration might account for the discrepancy. A much lower percentage of maternal deaths with known causes in Kilmarnock were not linked to a live birth: only 25 per cent. It is unclear why this might be, but if we were to assume that around 50 per cent of maternal deaths were not associated with a live birth, the final adjusted maternal mortality rate would be as high as 159 deaths per 10,000 births.

6) Adjustment for background mortality:

So far we have been working with a definition of maternal mortality which treats all deaths during pregnancy or within six weeks of childbirth as maternal. This is known as 'pregnancy related mortality'. However it is not strictly comparable to maternal mortality as some of the deaths in the indirect category will have been entirely unrelated to pregnancy - these women will have died even if they had not been pregnant or recently delivered. We calculated background mortality as the non-maternal number of deaths per year among women of childbearing age (15-44 years), per 10,000 women in the same age group. We then worked out an expected number of deaths in the six weeks after a birth, among women who had given birth in a year. At prevailing levels of background mortality, we would expect 42 non-maternal deaths to childbearing women in the six weeks after childbirth in Kilmarnock, and 12 on Skye, over the 40 year period. We assume these would all have been classed as indirect mortality.

Appendix 2: The certification practices of individual doctors

The vast majority of maternal deaths in Kilmarnock in the late nineteenth century were certified by a doctor, yet it has been shown above that over half (52 per cent) of them were misclassified or inadequately described, so that they were not counted among maternal deaths in official statistics. It is interesting to examine patterns of certification by individual doctors to see if particular doctors were more likely to hide maternal deaths and if so, whether there were systematic patterns among doctors. Table A.5 shows, for doctors who certified at least six maternal deaths, numbers of direct and indirect maternal deaths, and the percentage of each of these that were 'hidden' by the registrar and the doctor. Numbers of puerperal fever deaths are also shown separately, with the percentage of the puerperal fever deaths 'hidden' by registrar and doctor, and as a percentage of each doctor's direct deaths. Numbers of maternal deaths are very small, so the table should be seen as suggestive rather than conclusive: doctors who certified more than twenty maternal deaths are shown in bold. The date of each doctor's first qualification is shown, as an indicator of his medical cohort, and the dates he was visible within the database, practising in Kilmarnock (NB the latter dates may be left-censored in 1861 or right-censored in 1901). The doctors are ordered in descending order of the date of first qualification.

Table A.5: Certification of maternal deaths by individual doctors, Kilmarnock 1861-1901.

					direct maternal deaths			puerperal fever				indirect mortality		
Christian names	Surname	year of 1st qualification	first seen in K	last seen in K	no	% hidden by registrar	% hidden by doctor	no	% hidden by registrar	% hidden by doctor	PF as % direct	no	% hidden by registrar	% hidden by doctor
James M	Aitken	1828	1861	1872	2	0.0	0.0	2	0.0	0.0	100.0	4	0.0	75.0
John	Thomson	1829	1861	1865	5	0.0	100.0	4	0.0	100.0	80.0	1	0.0	100.0
John	Borland	1831	1861	1891	5	20.0	20.0	2	0.0	0.0	40.0	6	0.0	100.0
**Donald**	**Macleod**	**1850**	**1861**	**1901**	27	3.7	25.9	20	5.0	25.0	74.1	28	3.6	89.3
**Alexander**	**Marshall**	**1851**	**1861**	**1894**	74	5.4	14.9	55	3.6	7.3	74.3	41	4.9	92.7
**James**	**Rankin**	**1857***	**1869**	**1901**	24	8.3	33.3	12	0.0	58.3	50.0	16	12.5	87.5
**James**	**Mcalister**	**1858***	**1866**	**1900**	23	8.7	4.3	12	0.0	0.0	52.2	14	28.6	64.3
Alexander Wm	Macfarlane	1866*	1875	1887	9	0.0	0.0	7	0.0	0.0	77.8	1	0.0	100.0
William	Frew	1872	1888	1901	15	6.7	13.3	12	0.0	16.7	80.0	4	50.0	50.0
**John C**	**McVail**	**1873**	**1874**	**1891**	14	7.1	0.0	9	11.1	0.0	64.3	8	12.5	62.5
William	Mcalister	1879	1885	1901	5	0.0	0.0	3	0.0	0.0	60.0	5	80.0	20.0
**Wm. Aitken**	**MacLeod**	**1881**	**1882**	**1897**	26	0.0	65.4	24	0.0	66.7	92.3	12	0.0	75.0
David	Lawrie	1881	1887	1901	5	0.0	60.0	3	0.0	100.0	60.0	6	16.7	50.0

Notes: Doctors with more than 20 maternal deaths are shown in bold.
* indicates Licentiate of Midwifery qualification.

It is clear that there are quite large differences between doctors in the numbers of maternal deaths they certified per year. This is in part due to the length of the 'window' in which they were observed practising in Kilmarnock, but is also likely to be connected to the balance of their practice. Those with large numbers should not be assumed to be incompetent obstetricians or poor doctors: it is as likely that these were the doctors for whom maternity cases figured more highly among their clientele. The obituaries of some of these doctors mention this explicitly: James McAlister, for example, was said to have been ‘particularly successful in obstetric practice’ (BMJ 1900), and information about the obstetric practice of others can be gleaned from articles and letters to medical journals. For example in 1855 John Thomson published a statistical report about his 3300 deliveries in the previous 15 years, and a further report in 1864, by which time his tally had risen to 5000 cases (Thomson 1855, 1864). Others contributed isolated reports of interesting midwifery cases, some with maternal deaths and some with deaths averted, to local and national medical journals (McLeod 1857; MacDonald 1893, Arbuckle 1885, McLeod 1889). Midwifery was clearly a large part of general practice for many of the Kilmarnock doctors: John Thomson averaged 143 deliveries a year over a 35 year career and Donald McLeod about 175 a year over a 40 year career (Thomson 1864; McLeod 1889). These delivery rates would be high for a midwife: in 1915 Janet Lane-Claypon considered that 150 cases a year was ‘as many as one midwife can reasonably undertake’, so it is highly likely that the women were cared for during labour by a monthly nurse or handy-woman, with the doctor arriving only for the delivery (Lane-Claypon 1915). It is even possible that the doctors did not actually deliver all the births themselves (Leap and Hunter 1993), but given that they were booked for the delivery it is likely that they certified any maternal deaths which resulted. We do not know how many women each doctor delivered in each year, so we cannot compare maternal mortality ratios for each doctor; instead our focus is on investigating how different doctors certified maternal deaths.

Overall 28 per cent of direct deaths were hidden, but there was considerable variation between individual doctors. It was not until the Medical Act of 1886 that all medical training had to include obstetrics, although the Medical Act of 1858 had made it possible and some medical schools had instituted compulsory obstetric training from even earlier dates, such as Edinburgh in 1833 (Reid 2012). There is little evidence that those trained in midwifery were any better at certifying direct maternal deaths: although two of the doctors with Licentiates in Midwifery (James McAlister and Alexander MacFarlane) rarely failed to mention childbirth, this is not true for the third (James Rankin). Puerperal fever was the primary cause of direct maternal death, amounting to 71 per cent of

all the direct deaths to these doctors. If doctors were feeling sensitive about their maternal deaths, and recognised their potential role in transferring puerperal fever, they might have been more likely to 'hide' puerperal fever than other direct deaths. While this might be true for some doctors (such as James Rankin), it is not true for all, and several doctors provided clear causes for all the puerperal fever deaths we can also identify.

Perhaps then, it is more likely to be sloppy certification rather than deliberate obfuscation which hides the deaths, and this possibility is lent weight by the case of John Thomson. Only the final four years of Dr Thomson's career are captured in the data set, but he had been practising in Kilmarnock for many previous years, and leaves for posterity two published articles in the Glasgow Medical Journal relating to his extensive obstetric practice (Thomson 1855, 1864). Information in these papers indicates that his parturient patients died at a rate of about 40 per 10,000 deliveries, just a little lower than that in Kilmarnock as a whole from 1861 to 1900. Although we only have four years of his deaths, we can identify five direct (four of them from puerperal fever), and one indirect, maternal deaths in that time. This is a little higher than expected given his average deliveries per year, but may be the product of small number fluctuation. His maternal deaths are shown in table A.6.

The detailed texts of his articles suggest that he was skilled at midwifery, knowing when to let a labour progress naturally and when to intervene, and the fact that he admitted in print to so many instances of maternal mortality, 60 per cent of which he himself attributed to puerperal fever, suggests that he was not actively trying to hide his maternal deaths. In his articles he attributed five maternal deaths between 1855 and 1864 to puerperal fever, but none of the deaths he certified that we can find in the records would have been classified by clerks at the time as maternal, even though it is highly likely that those which he certified as being from 'peritonitis' or 'fever' were indeed puerperal fever. Because Scottish doctors did not have to hand the death certificate to the family, but gave it straight to the registrar, he was presumably not dissembling for any reason. Rather we suggest that he knew these deaths were 'puerperal peritonitis' and that he was simply not being specific enough in the type of peritonitis, failing to realise that there was then no reason for the coding clerks to know that the death was maternal. In contrast to John Thomson, James McAlister made the maternal nature of deaths in childbirth clear on the death certificates he wrote: only one direct maternal death was given a cause which did not mention childbirth in some way.

Table A.6: John Thomson's maternal deaths, Kilmarnock 1861-1865

days since birth of child	length of last illness	original cause of death given by Thomson	probable contemporary causal group	new causal group
3	5 & 4 days	diarrhoea, peritonitis	diarrhoeal diseases	puerperal fever
4	3 days	infammation of bowels	diarrhoeal diseases	indirect maternal deaths
5	3 days	peritonitis	peritonitis	puerperal fever
8	7 days	peritonitis	peritonitis	puerperal fever
9	2 days	fever	infectious diseases	puerperal fever
31	4 weeks	repeated haemorrhage	other causes	haemorrhage

Appendix Bibliography

Arbuckle, J.H. 1885. Complete cure of inversion of the uterus, *The Lancet* Dec 26 1885: 1183.

BMJ. 1900. Obituary: James McAlister, *BMJ* April 7 1900: 882.

Kippen, Rebecca. 2005. Counting maternal deaths in the nineteenth century: The case of Tasmania, *Historical Methods* 38(1): 14-25.

Lane-Claypon, J. 1915. The economic aspect of midwifery, in Forty-fourth annual report of the Local Government Board, 1914-15. Supplement in continuation of the medical officer of the board for 1914-15 containing a report on maternal mortality in connection with childbearing and its relation to infant mortality. London: HMSO, Cd. 8085: 85-104.

Leap, N. and B. Hunter. 1993. The midwife's tale: An oral history from handywoman to professional midwife. London: Scarlet Press.

Loudon, Irvine.1992. Death in Childbirth: An International Study of Maternal Care and Maternal Mortality, 1800-1950. Oxford: Clarendon Press.

MacDonald, David. 1893. Triplets. *British Medical Journal* Feb 18 1893: 392.

McLeod, D. 1857. Case of complete retroversion of the uterus, where the mother died, undelivered, in the tenth month of her pregnancy. *Glasgow Medical Journal* Volume: 410-414.

McLeod, Donald. 1889. The forceps and the perineum, *British Medical Journal* March 23 1889: 695-6.

Reid, Alice. 2012. Birth attendants and midwifery practice in early twentieth century Derbyshire, *Social History of Medicine* 25: 380-99.

Ronsmans, Carine and Wendy Graham. 2006. Maternal mortality: who, when, where and why. *The Lancet* 368:1189-1200.

Thomson, John. 1855. Statistical report of three thousand three hundred cases of obstetricy. *Glasgow Medical Journal* 3(10):129-150.

Thomson, John. 1864. Statistical report of five thousand cases of obstetricy. *Glasgow Medical Journal* VOLUME: 27-36.

Woods, R. & Shelton, N. 1997. *An Atlas of Victorian Mortality*. Liverpool: Liverpool University Press.
